# Non-Coding RNAs in Sepsis-Associated Acute Kidney Injury

**DOI:** 10.3389/fphys.2022.830924

**Published:** 2022-04-08

**Authors:** Yanna Chen, Huan Jing, Simin Tang, Pei Liu, Ye Cheng, Youling Fan, Hongtao Chen, Jun Zhou

**Affiliations:** ^1^ Department of Anesthesiology, The Third Affiliated Hospital, Southern Medical University, Guangzhou, China; ^2^ Department of Anesthesiology, The First People’s Hospital of Kashgar, Xinjiang, China; ^3^ Department of Anesthesiology, The Second People’s Hospital of Panyu, Guangzhou, China; ^4^ Department of Anesthesiology, Guangzhou Eighth People’s Hospital, Guangzhou Medical University, Guangzhou, China

**Keywords:** sepsis, acute kidney injury, miRNA, lncRNA, circRNA

## Abstract

Sepsis is a systemic inflammatory response caused by a severe infection that leads to multiple organ damage, including acute kidney injury (AKI). In intensive care units (ICU), the morbidity and mortality associated with sepsis-associated AKI (SA-AKI) are gradually increasing due to lack of effective and early detection, as well as proper treatment. Non-coding RNAs (ncRNAs) exert a regulatory function in gene transcription, RNA processing, post-transcriptional translation, and epigenetic regulation of gene expression. Evidence indicated that miRNAs are involved in inflammation and programmed cell death during the development of sepsis-associated AKI (SA-AKI). Moreover, lncRNAs and circRNAs appear to be an essential regulatory mechanism in SA-AKI. In this review, we summarized the molecular mechanism of ncRNAs in SA-AKI and discussed their potential in clinical diagnosis and treatment.

## Introduction

Sepsis is a common cause of acute conditions and death in patients with community-acquired and nosocomial infections (Wacker et al., 2013). The Third International Consensus (Sepsis-3) Definitions published in 2016 defines sepsis as life-threatening organ dysfunction caused by a dysregulated host response to infection (Singer et al., 2016). This condition can lead to various organ damage, such as cardiac insufficiency, acute respiratory distress syndrome, diffuse intravascular coagulation, and acute kidney injury (AKI).

AKI is a clinical syndrome caused by the rapid decline of renal function, associated with the risk of developing chronic kidney disease (CKD) and end-stage kidney disease (ESKD) (Bellomo et al., 2012). The incidence of death in AKI patients has been reported to be increased 5.5-fold compared with non-AKI patients (Chertow et al., 2005). The main causes of AKI are sepsis, obstruction, drugs, radiocontrast, and surgery; yet, sepsis is considered as one of the most common causes (Chertow et al., 2005; Uchino, 2005). In the intensive care unit (ICU), sepsis-associated AKI (SA-AKI) is diagnosed in up to 47% of cases and is a dominant public health concern associated with increased mortality and increased progression to CKD (Keir and Kellum, 2015). Effective and early detection, as well as proper treatment for patients with SA-AKI, are still missing. In addition, the molecular mechanisms of SA-AKI have not yet been fully explored.

The development of SA-AKI is a complex pathological process. Different from other types of AKI, renal blood perfusion in SA-AKI is not reduced, and even increased. And the culprit of SA-AKI is excessive inflammatory response (Kellum and Prowle, 2018). Over recent years, many studies have reported on the possible mechanism of SA-AKI, including the regulatory role of ncRNAs in the molecular mechanism of SA-AKI (Langenberg et al., 2014; Qin et al., 2020). The ncRNA family is transcribed from the genome without or with a small amount of protein-encoding potential that has multiple functions. ncRNAs can be divided into two main types: basic primary structure and regulatory ncRNAs. Basic structure ncRNAs exert a role similar to housekeeping genes in translation and splicing, including ribosomal RNA (rRNA), transfer RNA (tRNA), small nuclear RNA (snRNA), and so on. Regulatory ncRNAs mainly participate in the modification of other RNAs, including microRNA (miRNA), long non-coding RNA (lncRNA) and circular RNA (circRNA), piwi-interacting RNA (piRNA), small interfering RNA (siRNA), and so on. ncRNAs are involved in gene transcription, RNA processing, and post-transcriptional translation but also in the epigenetic regulation of gene expression (Cech and Steitz, 2014). Existing studies have demonstrated that ncRNAs might regulate gene expression by inhibiting target gene translation and inducing the degradation of target gene mRNA to regulate inflammation, programmed cell death, and oxidative stress in SA-AKI (Liu et al., 2020a; Luo et al., 2020a; Shi et al., 2020a). In the present review, we summarized the alteration, function, predictive value, and therapeutic potential of ncRNA (mainly miRNA, lncRNA, circRNA) in SA-AKI.

### The Role of ncRNA in SA-AKI

**FIGURE 1:**
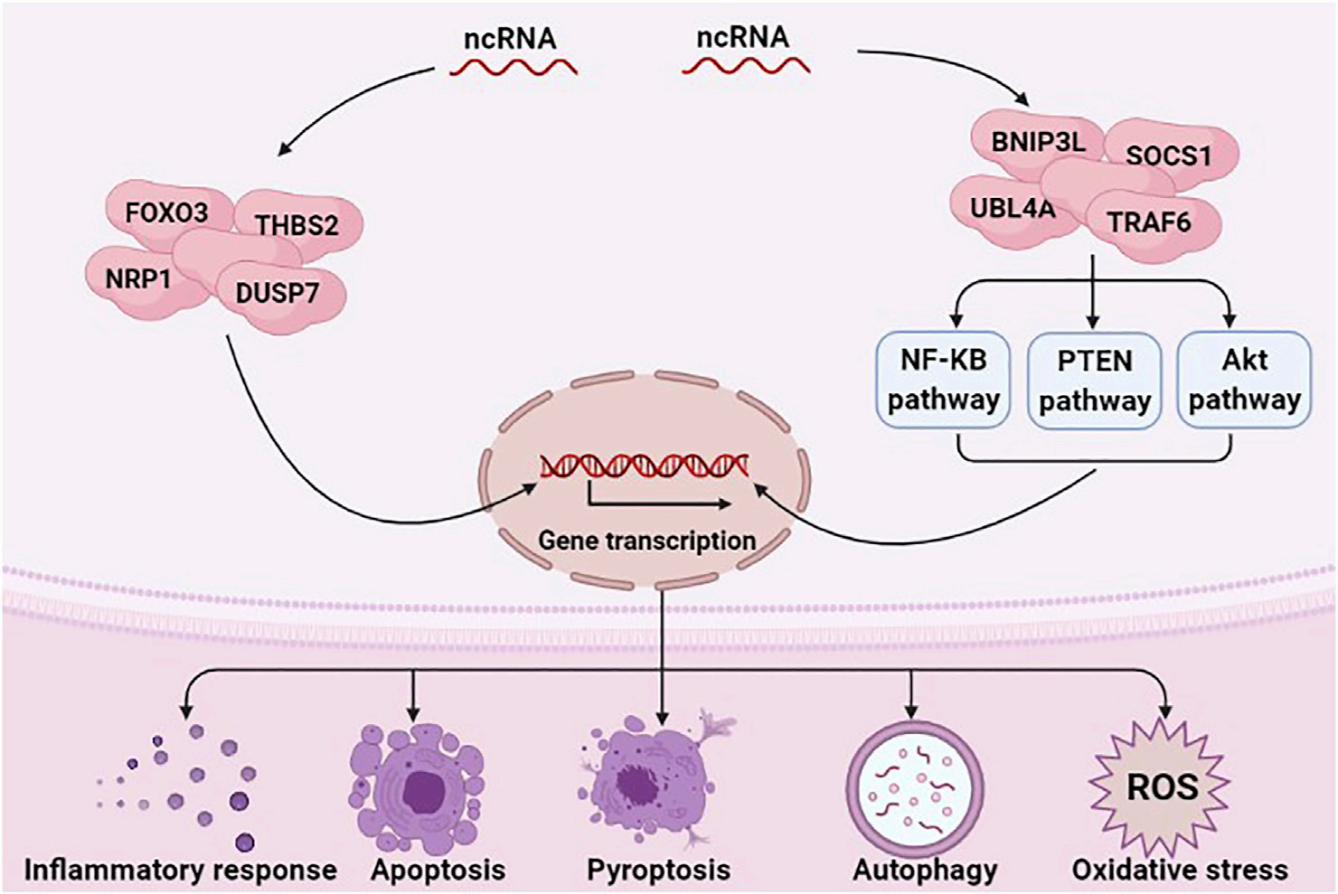
ncRNAs are involved in the pathological mechanism of SA-AKI. ncRNAs, including miRNAs, lncRNAs, and circRNAs, have a critical role in regulating inflammation response, programmed cell death, and oxidative stress in SA-AKI by regulating the expression of target genes or regulating various signaling pathways.

ncRNAs exert a regulatory role in many diseases, including sepsis (Yong, 2020) and AKI (Yu et al., 2016). Numerous studies have indicated that ncRNAs regulate inflammation, programmed cell death, and oxidative stress in SA-AKI by regulating the expression of target genes or regulating various signaling pathways (Figure 1) (Chen et al., 2018a; Wang et al., 2020a). E.g., miRNAs can aggravate or alleviate SA-AKI by regulating the expression of forkhead box O3 (FOXO3), neuropilin 1 (NRP1) and thrombospondin 2 (THBS2) (Shen et al., 2019a; Luo et al., 2020a; Wang et al., 2020b). miR-155 can activate Janus activated kinase (JAK)-signal transducer and activator of transcription (STAT) signaling pathway via negatively regulating suppressor of cytokine signaling 1 (SOCS1) (Ren et al., 2017).

**FIGURE 2:**
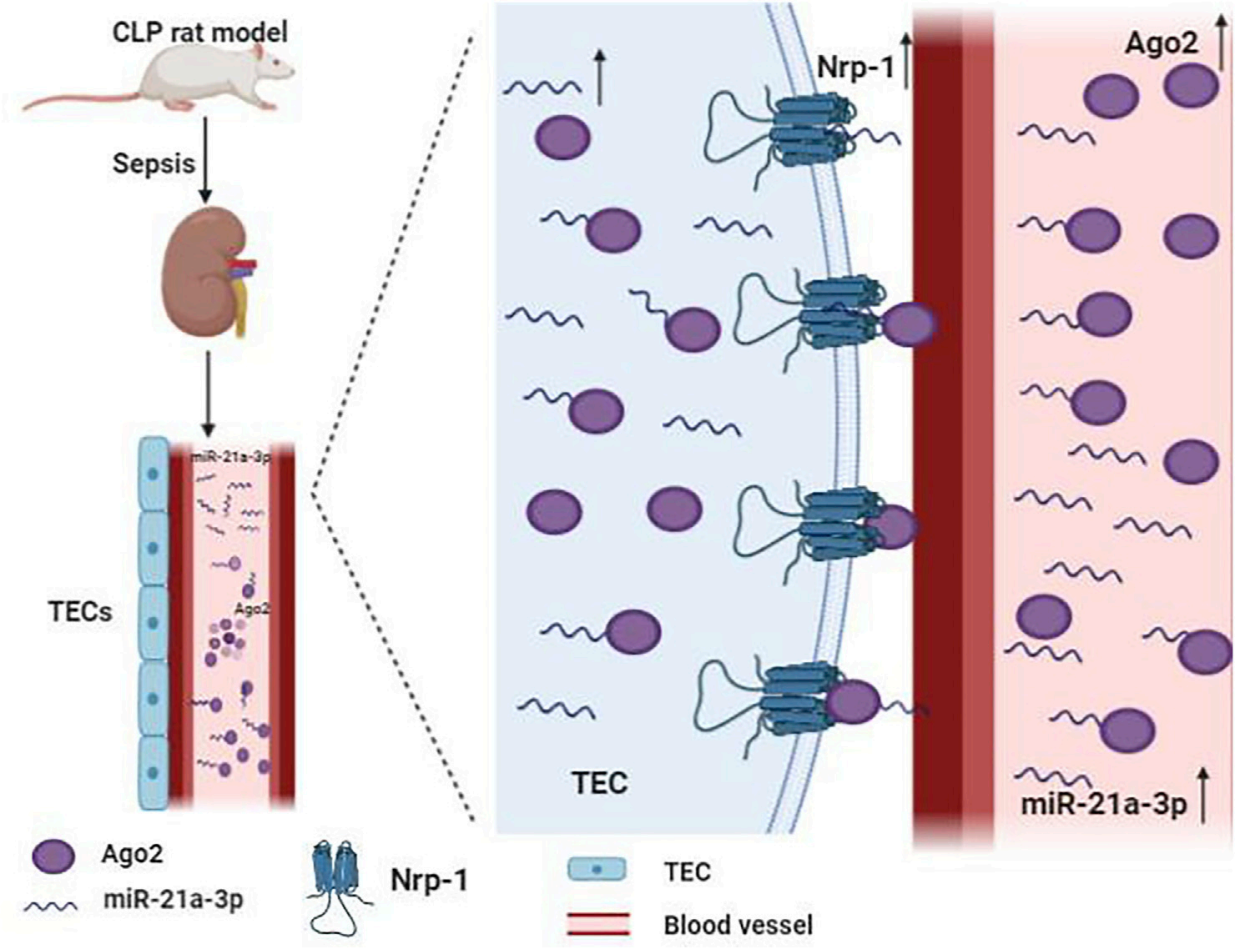
Mechanism of the accumulation of miR-21a-3p in tubular epithelial cells (TECs) during sepsis. miR-21a-3p stably exists in blood and urine by binding to argonaute2 (ago2). Neurocorin-1 (Nrp-1) acts as cell “cargo” transporting extracellular molecules, such as miR-21a-3p and miR-21a-3p-ago2, into cells.

miR-21 is the most frequently studied miRNA in SA-AKI. It is ubiquitously expressed in many organs like the heart and kidney in mammals (Chuppa et al., 2018; Song et al., 2018). Previous studies have suggested that miR-21 is a critical regulator of inflammation, energy metabolism, and cancer biology (Gomez et al., 2015; Zheng et al., 2017). Moreover, miR-21 is believed to be a regulatory molecule of SA-AKI involved in the pathological process of kidney fibrosis and mediated podocyte apoptosis (Song et al., 2018; Chen et al., 2018b; Wei et al., 2020). Emerging evidence indicated that the expression of miR-21 is upregulated in patients with SA-AKI (Wei et al., 2020). miR-21 stably exists in blood and urine by binding to argonaute2 (ago2), an essential binding protein of miRNAs. Neuropilin-1 (Nrp-1), a receptor that can bind various ligands via its extracellular part that consists of several domains, can function as a cell “cargo” by transporting extracellular molecules, such as miR-21 into cells. miR-21a-3p is internalized via Nrp-1 and ago2 that are important mechanisms for the accumulation of miR-21a-3p in tubular epithelial cells (TECs) during SA-AKI (Figure 2) (Zou et al., 2020). It has been reported that overexpression of miR-21 aggravates renal dysfunction and promotes cell apoptosis during lipopolysaccharide (LPS)-stimulated AKI (Lin et al., 2019a; Wei et al., 2020). Lin *et al* found that miR-21 has a critical role in apoptosis and is involved in cellular metabolic processes such as lipid metabolism and cell cycle arrest via AKT/CDK2-FOXO1 pathway in SA-AKI (Lin et al., 2019a). However, some studies have reached different conclusions. Few studies revealed that miR-21 exerts an anti-apoptosis effect, while its overexpression can inhibit pro-apoptotic signaling pathways phosphatase and tension homolog (PTEN)/Akt and PDCD4/nuclear factor-κB (NF-κB) in the kidney, thereby alleviating kidney injury caused by sepsis (Jia et al., 2015; Fu et al., 2017; Yang et al., 2018; Pan et al., 2019). It has also been demonstrated that xenon and remote ischemic preconditioning can protect against SA-AKI by upregulating the expression of miR-21 (Jia et al., 2015; Pan et al., 2019). The reasons for these discrepant findings are unclear, and may be related to the usage of different models. Thus, more evidence is needed to clarify the role of miR-21 in SA-AKI.

miR-22 is a frequently analyzed miRNA in the context of SA-AKI. miR-22 has been confirmed to have a critical role in many kidney diseases (Fan et al., 2016; Zhang et al., 2018b). Studies have found that miR-22 is down-regulated in patients with SA-AKI (Ge et al., 2017); the same results were obtained in the animal models constructed by cecal ligation and puncture (CLP) and LPS-treated cells (Wang et al., 2020a). Furthermore, the overexpression of miR-22 can reduce the expression of inflammatory factors (IL-1β, IL-6, and TNF-α) and NO, as well as significantly reduce cell apoptosis via PTEN/high-mobility group Box 1 (HMGB1) and TLR4/NF-κB pathway, thereby alleviating the AKI caused by sepsis (Shen et al., 2018; Shen et al., 2019b; Wang et al., 2020a). Besides, miR-22 negatively regulates the expression of apoptosis-inducing factor mitochondrion-associated 1 (AIFM1), and AIFM1 knockdown significantly ameliorates LPS-induced renal cell apoptosis *in vitro* (Zhang et al., 2020a). These findings imply that upregulation of miR-22 acts as a defense mechanism to decrease inflammatory response and apoptosis. To sum up, miR-22 may be a potential target to treat SA-AKI.

The expression of lncRNA NEAT1 has been confirmed to be significantly higher in ischemia-induced AKI patients compared to the healthy control group (Jiang et al., 2019a). Meanwhile, the expression levels of NEAT1 were also obviously higher in the patients with sepsis than in healthy controls. Furthermore, NEAT1 has been shown to be positively correlated with the severity of AKI, the increased disease risk, and the unfavorable prognosis in sepsis patients (Chen et al., 2018a; Huang et al., 2018). Besides, the expression of NEAT1 was also upregulated in SA-AKI model rats constructed by CLP and LPS-stimulated cells (Wang et al., 2020c). These results suggest that NEAT1 could be used as a useful additive biomarker for the diagnosis of sepsis. Functional studies revealed that suppression of NEAT1 alleviates the inflammatory response and cell apoptosis in LPS-treated rat mesangial cells (RMCs) by targeting miR-204 and modulating the NF-κB pathway (Chen et al., 2018a). Another study suggested that the expression of NEAT1 could improve renal function and ameliorate LPS-induced inflammatory responses and apoptosis via miR-27a-3p/TAB3 axis (Wang et al., 2020c). In LPS-treated mouse RAW264.7 macrophages, knockdown of NEAT1 alleviated LPS-induced inflammation by promoting macrophage M2 polarization via miR-125a-5p/TRAF6/TAK1 axis (Wang and Guo, 2020). These studies indicated that NEAT1 might be a target for the treatment of patients with SA-AKI.

circRNA is also considered to be implicated in SA-AKI. circ_00114,428 were upregulated in SA-AKI serum specimens and LPS-induced HK2 cells, and circ_00114,428 knockdown attenuated SA-AKI by regulating cereblon (CRBN) expression via targeting miR-495-3p (He et al., 2021). circTLK1 contributed to SA-AKI by regulating inflammation and oxidative stress through the miR-106a-5p/HMGB1 axis. circTLK1 and miR-106a-5p may be employed as the potential targets for the treatment of SA-AKI (Xu et al., 2021). circ_0091702 was downregulated in SA-AKI patients and LPS-induced HK2 cells. It could attenuate SA-AKI via the miR-545-3p/THBS2 axis, indicating that circ_0091702 might be an important factor for relieving SA-AKI (Tan and Bei, 2021). Evidence indicated that ncRNAs remarkably change in SA-AKI, while upregulation or downregulation of the expression of ncRNAs could change the prognosis of SA-AKI.

### ncRNAs Mediates Signaling Pathway in SA-AKI

The exact molecular mechanisms of SA-AKI are still unclear. However, existing studies have suggested that multiple signaling pathways contribute to the pathogenesis of SA-AKI, such as NF-κB, PTEN and Akt (Wang et al., 2020a; Zhong et al., 2020). Understanding the signaling pathways through which sepsis triggers AKI may promote new therapeutic approaches to prevent or reverse AKI.

### NF-κB Signaling Pathway

**FIGURE 3:**
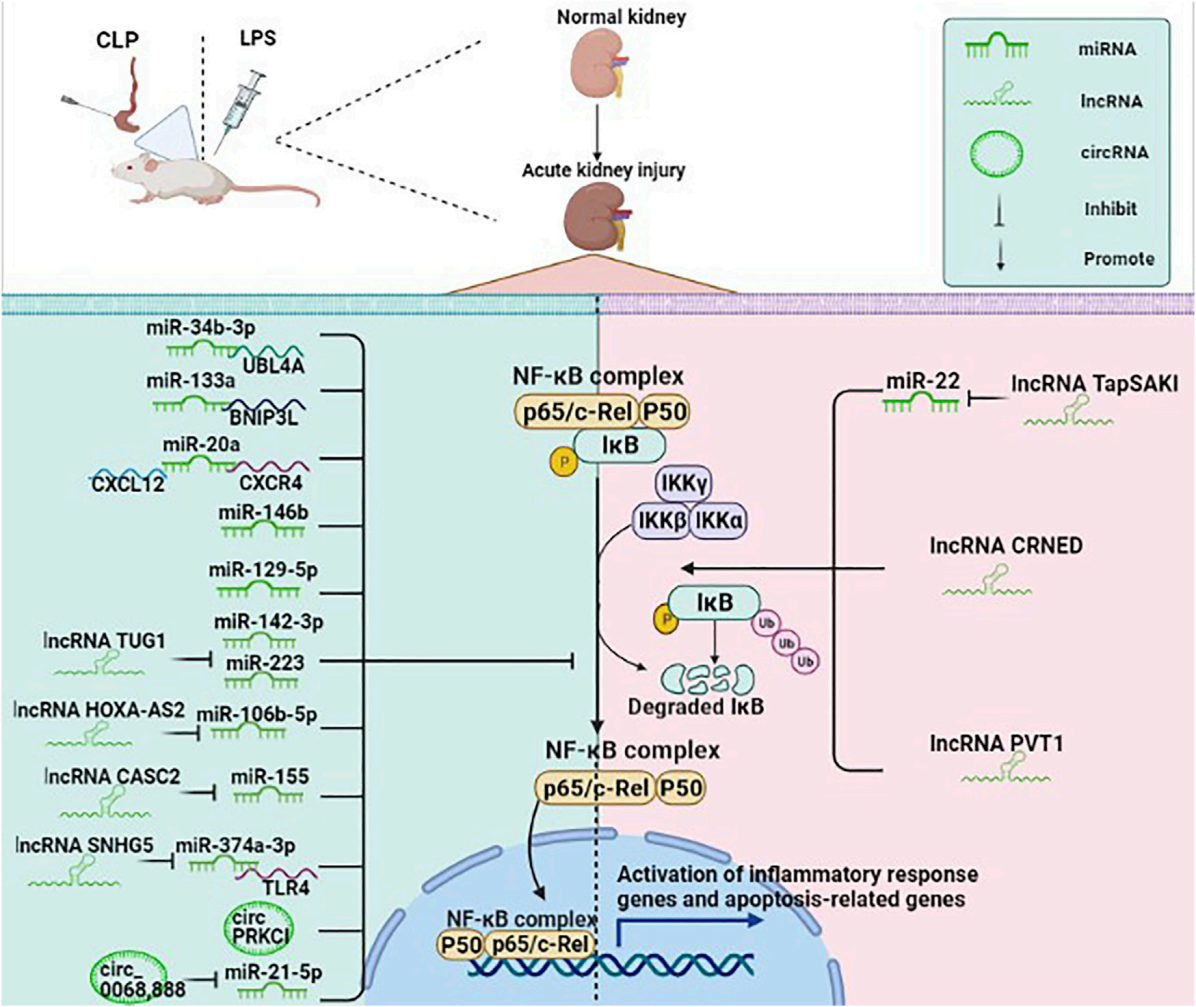
ncRNAs (mainly miRNAs, lncRNAs, circRNAs) participate in the NF-κB pathway in SA-AKI. NF-κB is a vital transcription factor in the cytoplasm in an inactive state, composed of the dimeric p50/p65 and another inhibitory subunit IκB. When IκB is catalyzed by IκB kinase, it undergoes phosphorylation and degradation, causing NF-κB to rapidly activate, while the activated NF-κB enters the nucleus and binds to numerous gene promoters, regulating various pathological processes. ncRNAs are involved in the pathological process of SA-AKI by regulating the NF-κB signaling pathway.

NF-κB is a vital transcription factor in the cytoplasm composed of the dimeric p50/p65 and another inhibitory subunit IκB. When IκB is catalyzed by IκB kinase, it undergoes phosphorylation and degradation, causing NF-κB to activate rapidly. The activated NF-κB then enters the nucleus and binds to numerous gene promoters, regulating various pathological processes (Qin et al., 2020). NF-κB signaling pathway is an important signaling pathway in the process of inflammatory response and immune response, which can regulate apoptosis and stress response (Luo et al., 2020b). Growing evidence has indicated that ncRNAs are involved in the pathological process of SA-AKI by regulating the NF-κB signaling pathway (Figure 3). For example, miR-34b-3p can decrease the transcriptional activity of NF-κB by inhibiting ubiquitin-like protein 4A (UBL4A). The expression of miR-34b-3p is suppressed in the CLP mice model and the LPS-induced RMCs. Moreover, overexpression of miR-34b-3p could alleviate kidney tissue injury through downregulation of UBL4A/NF-κB (He et al., 2020a).

BCL2/adenovirus E1B interacting protein 3-like (BNIP3L) belongs to the Bcl-2 family and is a BH3-only pro-apoptotic factor. miR-133a can directly target the BNIP3L and then inhibit the NF-κB pathway, thereby inhibiting inflammation and apoptosis and ameliorating the renal function (Qin et al., 2020). Besides, previous studies have shown that the chemokine C-X-C motif ligand 12 (CXCL12)/C-X-C chemokine receptor type 4 (CXCR-4) axis could activate the NF-κB signaling pathway. miR-20a may exert anti-inflammatory and anti-apoptotic effects in LPS-stimulated HK-2 cells by inhibiting CXCL12/CXCR-4 and NF-κB signaling pathways (Zhang et al., 2020b). Human umbilical cord mesenchymal stem cell-derived exosomes (HucMSC-Ex) can decrease interleukin (IL)-1 receptor-associated kinase (IRAK1) level through the upregulation of miR-146b level, which suppress NF-κB activity, eventually alleviating SA-AKI and improving survival in mice with sepsis. HK-2 cells transfected with miR-146b inhibitor can significantly increase the translocation of NF-κB p-P65 to the nucleus, while HK-2 cells transfected with miR-146b mimic could lead to an opposite result, thus suggesting that miR-146b improves renal function by inhibiting NF-κB signaling pathway (Zhang et al., 2020c). miR-129-5p has been shown to reduce LPS-induced SA-AKI models *in vitro* and *in vivo* and exert a protective effect. miR-129-5p reduced the expression of nuclear NF-κB protein, thereby reducing podocyte apoptosis and inflammation (Huang et al., 2020a).

lncRNAs are involved in the NF-κB signaling pathway by regulating the miRNAs. A previous study suggested that lncRNA TrAnscript Predicting Survival in AKI (TapSAKI) was elevated in urine-derived sepsis (US)-induced kidney injury. It increased the expression of TLR4, p-p65, and caspase-3 by negatively regulating miR-22, promoting apoptosis and inflammatory response through the TLR4/NF-κB pathway (Shen et al., 2019b). Moreover, the expression of lncRNA taurine upregulated gene 1 (TUG1) is downregulated in patients with SA-AKI. The upregulation of TUG1 inactivates NF-κB signaling to mediate LPS-related cell damage by negatively regulating the miR-142-3p or miR-223 (Liu, 2019). A previous study suggested that lncRNA HOXA cluster antisense RNA 2 (HOXA-AS2) is downregulated in sepsis patients, CLP mouse models, and LPS-stimulated HK-2 cells while its overexpression alleviates SA-AKI by targeting miR-106b-5p and restrains the Wnt/β-catenin and NF-κB pathways (Wu et al., 2020). Another study has shown that the expression of lncRNA cancer susceptibility candidate 2 (CASC2) was significantly decreased in sepsis patients compared with healthy subjects, and the level of the CASC2 was negatively correlated with the severity of kidney damage. CASC2 inhibited inflammation, apoptosis, and oxidative stress by negatively regulating miR-155 and NF-κB pathways, thus suggesting that CASC2 could serve as a potential target for treating SA-AKI (Wang et al., 2020d). Evidence suggested that lncRNA small nuclear RNA host gene 5 (SNHG5) can combine with miR-374a-3p in SA-AKI, which inhibits NF-κB activity by targeting TLR4 (Wang et al., 2021a).

lncRNAs could also directly regulate the NF-κB pathway without regulating the expression of miRNA. lncRNA colorectal neoplasia differentially expressed gene (CRNDE) was significantly upregulated in mouse models constructed by LPS. Downregulation of CRNDE could effectively inhibit the activation of the TLR3/NF-κB pathway to alleviate SA-AKI (Sun et al., 2019). Huang *et al* found that increased expression of lncRNA plasmacytoma variant translocation 1 (PVT1) in LPS-induced inflammation injury in HK-2 cells promoted inflammatory factors and proteins p-IκBα and p-p65, while its inhibition reversed this process. This study indicated that PVT1 might regulate the NF-κB pathway, having a pivotal role in inflammation (Huang et al., 2017).

The studies on the role of circRNA in the NF-κB signaling pathway in SA-AKI are limited. circPRKCI has been shown to be significantly decreased in patients with urosepsis and LPS-treated HK2 cells. Moreover, the overexpression of circPRKCI could reduce the phosphorylation level of IκBα, and the expression of p50 and p65 in the nucleus, thus suppressing the NF-κB pathways and reducing inflammatory response (Shi et al., 2020a). Decreased hsa_circ_0068,888 expression was observed in LPS-stimulated HK-2 cells, while hsa_circ_0068,888 overexpression suppressed the activation of NF-κB pathway by negatively regulating miR-21-5p as shown by decreased p-p65 protein level and nuclear translocation of p65 (Wei et al., 2021).

All these studies demonstrated that ncRNAs participate in the activation of the NF-κB signaling pathway. Yet, the specific mechanism remains unclear.

### Phosphatase and Tensin Homolog

PTEN, a lipid and protein phosphatase with widespread action, is a tumor suppressor gene that participates in the pathophysiological process of multiple organ injury (Ning, 2004; Lan et al., 2012; Bhatt et al., 2015). Recent evidence has shown that PTEN expression is increased in patients with sepsis and animal models of sepsis (Sisti et al., 2018).

PTEN can adjust the abundance of miRNAs by influencing enzymes involved in miRNAs maturation. For example, active PTEN can increase the expression of miR-125b and miR-21 and decrease the expression of miR-155 in the human embryonic kidney (HEK) 293 cells by regulating the nuclear localization of Drosha-Dgcr8 (part of the miRNA-processing complex) (Sisti et al., 2018). Conversely, miRNA can also regulate the expression of PTEN in SA-AKI, thereby mediating the pathological process of SA-AKI. Pan *et al* found an increased expression of PTEN in the CLP mouse models and LPS-treatment cells. Contrary, the upregulation of miR-21 induced by limb remote ischemic preconditioning (rIPC) reversed this process, exerting anti-apoptotic and anti-inflammatory effects and alleviating SA-AKI (Pan et al., 2019). Moreover, it has also been found that a protective role of miR-22-3p in SA-AKI may depend on the repression of PTEN (Wang et al., 2020a). Besides, a miR-205 agonist could improve the pathological morphology in the SA-AKI models by inhibiting the HMGB1-PTEN signaling pathway (Zhang et al., 2019a).

lncRNAs participate in the development of SA-AKI by regulating the PTEN signaling pathway. It has been found that lncRNA TCONS_00016406 (termed lncRNA 6406) was significantly downregulated in the SA-AKI models, and lncRNA 6406 could attenuate SA-AKI through modulating the miR-687/PTEN signaling pathway (Liu et al., 2020a). lncRNA TapSAKI positively regulates PTEN, and the knockdown of TapSAKI improves renal function (Shen et al., 2019b).

In the sepsis model, ncRNAs could improve AKI by inhibiting the expression of PTEN. In summary, PTEN may be a key factor in the pathological process of SA-AKI and may become a therapeutic target in the future.

### Akt Signaling Pathway

Protein kinase B, also known as Akt, has been shown to be the encoding product of the retrovirus safety gene V-Akt, which plays an important role in cell survival and apoptosis. Growth and survival factors such as insulin can activate the Akt signaling pathway. The mammalian target of rapamycin (mTOR) is a downstream target of phosphatidylinositol 3 kinase (PI3K) and Akt pathways. Akt activates mTOR complex 1 (mTORC1) to inhibit autophagy. The Akt signaling pathway is involved in the course of SA-AKI (Zhao et al., 2020a).

Study have shown that during SA-AKI, miR-21-3p upregulates FOXO1 by directly targeting Akt and CDK2, and induces TECs cell cycle G1 arrest and apoptosis (Lin et al., 2019a). In CLP mice, miR-214 activates the Akt/mTOR pathway to inhibit autophagy in kidney tissues, suggested that miR-214 served a protective role against SA-AKI (Sang et al., 2021). In the SA-AKI model, the expression of miR-93 is significantly down-regulated, and miR-93 can reduce apoptosis and inflammatory response by activating Akt/mTOR signaling pathway, thus mitigate SA-AKI (Zhan et al., 2021). It is known from the above that ncRNA can reduce apoptosis or autophagy by activating the Akt signaling pathway, thus improving SA-AKI, which may be a new idea for the treatment of SA-AKI.

### Role of ncRNAs in Cellular Mechanisms in SA-AKI

Accumulating evidence suggests that ncRNAs are involved in cellular mechanisms of SA-AKI, such as excessive inflammatory response, apoptosis, autophagy, and oxidative stress (Lee et al., 2012; Rousta et al., 2018; Zhang et al., 2019b).

### Inflammatory Response

The inflammatory response is one of the crucial pathogeneses of SA-AKI. In sepsis, circulating toxins may act on endothelial cells, causing an overactivated state of the immune system. Endothelial cells, epithelial cells, neutrophils, macrophages, and lymphocytes produce large amounts of pro-inflammatory cytokines (such as TNF-α, IL-6, IL-1β, and IL-8), then intracellular pathways are activated, and more pro-inflammatory cytokines and chemokines are released (Verma and Molitoris, 2015). Excessive release of pro-inflammatory factors causes the damage of renal TECs, which in turn leads to AKI (Huang et al., 2015). The previous study has shown that the inhibition of inflammatory cytokine production could ameliorate the severity of LPS-induced kidney injury (Rousta et al., 2018). Moreover, relevant evidence showed that ncRNAs have an essential role in the inflammatory response of SA-AKI by regulating the production of inflammatory cytokines (Liu et al., 2020a).

Existing studies indicated that ncRNAs exert an anti-inflammatory effect in SA-AKI. For instance, when treating HK-2 cells with LPS, the expression of miR-20a and miR-942-5p was downregulated, and inflammatory cytokines were upregulated (Luo et al., 2020a; Zhang et al., 2020b). In septic rat models, increased expression of miR-22-3p and miR-191-5p could reduce the inflammatory response (Qin et al., 2019; Wang et al., 2020a). Some studies have shown that some miRNAs, lncRNAs, and circRNAs can suppress inflammation response by inhibiting the transcriptional activity of NF-κB, thereby alleviating SA-AKI (Liu, 2019; Ma et al., 2019; He et al., 2020a; Lu et al., 2020a; Shi et al., 2020a; Wang et al., 2020d; Qin et al., 2020). It has also been reported that propofol can upregulate the expression of miR-290-5p, thereby reducing the expression of inflammatory cytokines to attenuate SA-AKI (Zheng et al., 2018). lncRNA HOX transcript antisense RNA (HOTAIR) overexpression in sepsis rats could reduce the IL-1β and TNF-α to improve renal function (Jiang et al., 2019b). A novel lncRNA TCONS_00016406 significantly reduced inflammation by targeting the miR-687/PTEN axis to reduce the inflammatory cytokines (including IL-1β, TNF-α, and IL-18) in SA-AKI (Liu et al., 2020a). The differentiation level antagonizing nonprotein coding RNA (DANCR) was markedly decreased in AKI patients and LPS-treated HK-2 cells, and overexpression of DANCR could suppress inflammatory response and cell apoptosis by regulating miR-214 (Zhao et al., 2020b). Also, previous studies have shown that circ-Ttc3 and circVMA21 reduce inflammation caused by SA-AKI in CLP rat models (Shi et al., 2020b; Ma et al., 2021).

ncRNAs can aggravate kidney damage by increasing the inflammatory response. miR-152-3p is upregulated in patients with SA-AKI and positively correlated with serum creatinine (SCr), blood urea nitrogen (BUN), IL-1β, and TNF-α. Downregulation of miR-152-3p could alleviate inflammatory response (Ma et al., 2020). miR-106a is also upregulated in the serum of sepsis patients. The same results were observed in CLP mouse models and LPS-treated TCMK-1 cells (Shen et al., 2019a). Besides, miR-106a was negatively regulated by lncRNA HOXA cluster antisense RNA 2 (HOXA-AS2), while its suppression could decrease LPS-induced inflammation factor level *in vitro* (Shen et al., 2019a; Wu et al., 2020). The expression of miR-128-3p and miR-155 was also evidently upregulated in renal tissues in SA-AKI mouse models while their inhibition alleviated renal injury, markedly decreasing the inflammatory cytokines (Ren et al., 2017; Wang et al., 2020b). miR-107 was dramatically increased in the circulating endothelial cells (CECs) of septic AKI patients compared with non-septic AKI and septic non-AKI patients, and miR-107 increased TNF-α secretion by targeting dual-specificity phosphatase 7 (DUSP7) in endothelial cells (Wang et al., 2017). Moreover, a previous study suggested that lncRNA TapSAKI promotes the inflammatory response by negatively regulating miR-22 (Shen et al., 2019b). lncRNA CRNDE was upregulated in patients with sepsis and SA-AKI models, and CRNDE overexpression markedly boosted inflammatory cytokine levels, including TNF-α, IL-6, IL-8, and IL-1β. At the same time, interfering with the expression of CRNDE could reverse the result (Wang et al., 2020e; Wu, 2020). lncRNA PVT1 has been shown to be upregulated in LPS-induced SA-AKI models, and PVT1 could mediate the release of pro-inflammatory cytokines (Huang et al., 2017). circ-FANCA (hsa_circ_0040994) is highly expressed in SA-AKI, and circ-FANCA silence alleviated LPS-induced HK2 cell injury, including proliferation inhibition and inflammatory response (Li et al., 2021a).

The inflammatory response is a critical pathological process of SA-AKI. ncRNAs have a regulatory role in this process by increasing the inflammatory response.

### Programmed Cell Death

Research suggested that SA-AKI is closely related to programmed cell death, a suicide protection measure initiated by gene regulation when cells are stimulated by internal and external environmental factors, including apoptosis, pyroptosis, and autophagy (Xue et al., 2019; Wang et al., 2020f; Ma et al., 2020).

#### Apoptosis

Apoptosis is a form of programmed cell death that depends on the activity of cysteine and cathepsin and can be triggered by exposure to stress stimuli as well as mitochondrial products. At present, it is thought that there is no significant apoptosis in the kidney during the progression of sepsis (Cao et al., 2019). However, the view that cell death caused by apoptosis during sepsis directly leads to microvascular dysfunction and organ failure, is still accepted by most people (Huang et al., 2020b). Some ncRNAs participate in the pathological process of SA-AKI via mediating cell apoptosis.

miRNAs have been confirmed to be involved in cell apoptosis. For example, miR-152-3p and miR-128-3p promote cell apoptosis to exacerbate renal dysfunction in sepsis (Wang et al., 2020b; Ma et al., 2020). miR-590-3p, miR-20a, miR-133a, and miR-942-5p exert an anti-apoptotic effect, while their overexpression can dramatically inhibit the expression of Bax (pro-apoptotic regulator) and increase the expression of Bcl-2 (anti-apoptotic regulator) in SA-AKI models (Ma et al., 2019; Luo et al., 2020a; Zhang et al., 2020b; Qin et al., 2020). miR-205 agonist has been shown to decrease apoptosis rate in the rat models of sepsis-induced renal injury, which suggested that miR-205 agonist could suppress cell apoptosis and alleviate renal injury (Zhang et al., 2019a).

lncRNA exerts anti-apoptotic or pro-apoptotic effects by regulating miRNA and signaling pathways. lncRNA TUG1 and lncRNA 6406 have an anti-apoptosis effect. They markedly mitigate LPS-induced cell damage by reducing cell apoptosis in SA-AKI, indicating that overexpressing TUG1 and 6406 may be an effective therapeutic strategy of SA-AKI (Liu et al., 2020a; Liu, 2019). lncRNA is also involved in the pro-apoptotic process of SA-AKI. It has been confirmed that interfering with the expression of lncRNA NEAT1 and lncRNA PVT1 could reduce apoptosis to facilitate the kidney cell injury caused by sepsis (Wang et al., 2020c; Huang et al., 2017). Moreover, lncRNA myocardial infarction-associated transcript (MIAT) may bind to miR-29a to promote cell apoptosis in LPS-stimulated cells (Zhang et al., 2019c). Through the miR-22/PTEN/TLR4/NF-κB pathway, lncRNA TapSAKI promotes cell apoptosis, thereby aggravating SA-AKI (Shen et al., 2019b). lncRNA ENST00000452391.1, also known as “sepsis-induced kidney injury associated transcript 1 (SIKIAT1)", acts as an apoptosis factor in SA-AKI and is upregulated in the peripheral blood samples of patients with SA-AKI. It could sponge miR-96-3p to facilitate Forkhead Box 1 (FOXA1) expression to promote cell apoptosis in LPS-stimulated HK-2 cells (Lu et al., 2020b).

lncRNA HOTAIR was upregulated in kidney tissues with sepsis and LPS-treated HK-2 cells. It was involved in cell apoptosis in SA-AKI. There are different conclusions regarding the role of HOTAIR in cell apoptosis. Shen *et al* suggested that HOTAIR promotes cell apoptosis by modulating miR-22/HMGB1 (Shen et al., 2018). Moreover, Jiang *et al* found that HOTAIR could inhibit the apoptosis of kidney cells in septic rats with AKI via inhibiting the miR-34a and increasing the Bcl-2 level. They also found that lncRNA HOTAIR overexpression can alleviate renal function (Jiang et al., 2019b). Yet, the exact role of HOTAIR in cell apoptosis in SA-AKI needs to be further confirmed.

The role of lncRNA CRNDE in regulating apoptosis in SA-AKI models is still controversial. Wang and others found that the down-regulation of lncRNA CRNDE promotes cell apoptosis and aggravates kidney injury by negatively regulating miR-181a-5p in sepsis (Wang et al., 2020g). In contrast, Wu *et al* suggested that the inhibition of lncRNA CRNDE could reduce cell apoptosis by inhibiting the NF-κB signaling pathway in SA-AKI (Sun et al., 2019; Wu, 2020).

Few studies in cell apoptosis in SA-AKI have focused on the circRNAs. circVMA21 has an anti-apoptotic role in SA-AKI, and overexpression of circVMA21 could inhibit apoptosis from ameliorating SA-AKI (Shi et al., 2020b). The expression of circRNA mitochondrial translation optimization one homolog (circMTO1) was significantly decreased in SA-AKI rat models, and LPS-stimulated RMCs and circMTO1 overexpression promoted cell viability, inhibited cell apoptosis. They also alleviated AKI by regulating miR-337 (Shi et al., 2020c). It has been reported that overexpression of circPRKCI increases cell viability and decreases cell apoptosis in LPS-treated HK-2 cells (Shi et al., 2020a). Knockdown of circ-FANCA could inhibit cell apoptosis and alleviate LPS-induced HK2 cell injury (Li et al., 2021a). The role of circRNAs in apoptosis and their specific mechanism in SA-AKI is still unclear. Additional studies are warranted to give a more profound insight into the role of circRNAs in apoptosis in SA-AKI.

#### Pyroptosis

Pyroptosis, also known as inflammatory cell necrosis, is manifested by the continuous expansion of cells until the cell membrane ruptures, which leads to the release of cell contents and activates a robust inflammatory response. Caspase-11, GSDMD, and inflammasomes can modulate pyrolysis and induce cell death under inflammatory and stress pathological conditions (Shi et al., 2017). It is key to study pyroptosis by detecting the expression of NLRP3, GSDMD and its related family members, as well as the cleavage of inflammatory caspases. Pyroptosis is a new type of programmed cell death that has been discovered and confirmed in the past decade, and its molecular mechanism of regulating the infectious diseases is a Frontier focus. However, how excessive pyroptotic signaling caused by infection triggers AKI remains to be understood. Evidence suggested that pyroptosis contributes to SA-AKI pathophysiology (Ye et al., 2019).

Although pyroptosis is a research hotspot related to sepsis, there is little evidence directly related to SA-AKI. For instance, lncRNA growth arrest specific transcript 5 (GAS5) has protective effects against SA-AKI via downregulating miR-579-3p to inhibit cell pyroptosis (Ling et al., 2021). lncRNA maternally expressed gene 3 (MEG3) promoted renal tubular epithelial pyroptosis by regulating the miR-18a-3p/GSDMD pathway in SA-AKI (Deng et al., 2021a). lncRNA PVT1 modulated nucleotide-binding oligomerization domain-like receptor protein 3 (NLRP3)-mediated pyroptosis in SA-AKI by targeting miR-20a-5p (Deng et al., 2021b). miR-21 is a critical positive regulator of the NF-κB pathway and NLRP3 inflammasomes in pyroptosis and septic shock (Xue et al., 2019). miR-30c-5p is down-regulated in patients with SA-AKI, septic mice, and HK-2 cells. miR-30c-5p inhibits the expression of pyroptosis-related marker caspase-11 and GSDMD by negatively regulating thioredoxin-interacting protein (TXNIP) (Li, 2020). Exosomal miR-93-5p also could regulate the TXNIP directly to influence the pyroptosis in renal epithelial cell (Juan et al., 2021). Studies suggested that miR-223-3p regulates endothelial cell pyroptosis by targeting NLRP3, and that miR-223-3p overexpression can partially reverse the cytotoxicity pyroptosis in HK-2 cells upon LPS stimulation by targeteing 3′UTR of NLRP3 and repressing its expression (Tan et al., 2020). Taken together, although the potential role of ncRNAs in pyroptosis has not been fully understood, emerging evidence shows the importance of ncRNAs in pyroptosis.

#### Autophagy

Autophagy is a process of engulfing one’s cytoplasmic proteins or organelles and coating them into vesicles, fusing with lysosomes to form autophagic lysosomes, and degrading the contents of the lysosomes, thereby fulfilling the metabolic needs of the cell itself and the renewal of specific organelles (Parzych and Klionsky, 2014). Studies have shown that ROS can trigger autophagy in mammalian cells and tissues in sepsis and AKI, and play a protective role by clearing pathogens, neutralizing microbial toxins, regulating cytokine release, reducing apoptosis oncotarget, and promoting antigen expression (Kaushal and Shah, 2016; Huang et al., 2019). However, related studies hold the opposite view that inhibition of autophagy can improve SA-AKI (Wang et al., 2020f), which may be related to the inhibition of autophagy can alleviate cell death when excessive autophagy. Currently, the role of autophagy in SA-AKI remains unclear and needs further study.

Autophagy is the pathological process of SA-AKI (Zhang et al., 2019b; Choi, 2020). ncRNAs participate in the pathological process of SA-AKI by regulating autophagy. It has been reported that miR-20a may promote AKI in septic rats by activating autophagy (Wang et al., 2020f). miR-526b could promote cell viability by inhibiting autophagy, and potentially targeting autophagy-associated gene 7 (ATG7) in SA-AKI (Liu et al., 2020b). miR-214 could alleviate AKI in septic mice by inhibiting the level of kidney autophagy through the regulation of the PTEN/AKT/mTOR pathway (Sang et al., 2021). Overexpression of TUG1 can reverse the expression of autophagy-related factors induced by LPS, like the increase of autophagy factors (LC3-II/I and beclin-1) and the decrease of p62 levels, thus inhibiting autophagy (Liu, 2019). In sepsis rats, lncRNA NKILA and the expression of autophagy-related proteins were significantly increased in kidney tissues, and lncRNA NKILA regulated the autophagy via PI3K/Akt pathway (Yang et al., 2020). At the same time, lncRNA NKILA knockdown represses cell autophagy in HK2 cells stimulated with LPS (Han et al., 2021). lncRNA SNHG14 was highly expressed in the plasma of sepsis patients with AKI and inhibited cell autophagy by negatively regulating miR-495-3p expression (Yang et al., 2021). ncRNA can affect the course of SA-AKI by affecting the expression of autophagy related factors, but the specific mechanism remains to be improved.

### Oxidative Stress

Oxidative stress refers to the imbalance of the generation and clearance of oxygen free radicals in the body or cells. It results in the accumulation of reactive nitrogen species (RNS) and reactive oxygen species (ROS), including superoxide (-O₂-), hydrogen peroxide (H₂O₂) and hydroxyl free radical (HO), thus causing oxidative damage. The release of large amounts of ROS into the cytoplasm leads to oxidative modifications of proteins and lipids that severely affect cell function. Antioxidant enzymes such as superoxide dismutase (SOD), catalase (CAT), peroxidase (POD), glutathione S-transferase (GST), glutathione peroxidase (GSH-PX), glutathione reductase (GR), and thioredoxin peroxidase (TPX) can reflect antioxidant activity. The content of antioxidant glutathione (GSH) can reflect the antioxidant capacity of the body. And the content of malondialdehyde (MDA) can reflect the level of lipid peroxidation. The clinical significance of oxidative stress in SA-AKI has been confirmed by multiple studies. The stimulation of LPS leads to increased oxidative stress in the kidney tissues of mice, causing overproduction of reactive oxygen species (ROS) and MDA, reducing SOD, GSH, and CAT activities in kidney tissues, leading to SA-AKI (Zhang et al., 2017).

ncRNAs regulate oxidative stress in SA-AKI by changing the expression of oxidative stress-related factors. The overexpression of lncRNA 6406 can mitigate SA-AKI via oxidative stress suppression, as indicated by the sharp increase in SOD1, GSH, and HO-1 levels (Liu et al., 2020a). lncRNA small nucleolus RNA host gene 14 (SNHG14) was involved in the oxidative stress of LPS-induced HK-2 cell and SNHG14 overexpression can enhance SA-AKI through oxidative stress (Shi, 2021). circVMA21 and circ-Ttc3 mitigated the oxidative stress caused by SA-AKI. An increased expression of circ-Ttc3 and circVMA21 could markedly decrease the levels of ROS and MDA and increase GSH and the activities of SOD and CAT, attenuating the SA-AKI (Shi et al., 2020b; Li et al., 2021b; Ma et al., 2021). Moreover, a study suggested that circ-FANCA knockdown could increase the level of SOD and decrease the level of MDA to attenuate LPS-induced HK2 cell injury by regulating OXSR1 expression (Li et al., 2021a). Dexmedetomidine increased the expression of antioxidant markers SOD and CAT and decreased the levels of MDA and SOD on rats with SA-AKI by upregulating miR-146a expression (Ni et al., 2020). Oxidative stress is an essential pathological process of SA-AKI, while ncRNAs enhance the ability of cells to resist oxidative stress by reducing ROS production, thereby improving kidney function.

## Clinical Applications of ncRNA in SA-AKI

ncRNAs have become a potential biomarker for SA-AKI detection. Moreover, regulation of their expression may provide new approaches for the treatment of SA-AKI.

### Predictive Value

The expression of specific miRNAs is related to the severity and prognosis of the disease (Ge et al., 2017; Lin et al., 2019b). A clinical study showed that urinary miR-26b level is significantly increased in patients with SA-AKI and may be used for distinguishing between AKI sepsis from non-AKI sepsis with a sensitivity of 90.8% and specificity of 75.0%. Moreover, the urinary miR-26b level is closely related to the mortality of patients, which suggests that the urinary miR-26b may become the potential biomarker for SA-AKI (Zhang et al., 2018a). Through sample collection and statistical analysis, Huo *et al* found that miR-29a and miR-10a-5p were positively correlated with the expression of Scr, Cystatin C (Cys-C), and kidney injury molecule 1 (KIM-1), which resulted as independent risk factors for death in patients with sepsis. Furthermore, the auROC value of the combined detection of miR-29a and miR-10a-5p was more effective than that of the single detection of miR-29a, miR-10a-5p, Cys-C, Scr, and KIM-1, which indicated that miR-29a and miR-10a-5p have good predictive value in assessing the mortality of patients with sepsis (Huo et al., 2017). In septic mice following LPS or CLP treatment, serum and urinary miR-452 increased early before detectable renal dysfunction or tissue damage, and in septic patients, serum and urinary miR-452 levels were significantly higher in patients with AKI than in those without AKI. Moreover, the sensitivity of urinary miR-452 in detecting AKI in septic patients was significantly higher than that in urinary tissue inhibitor metalloproteinase-2 [TIMP2]* insulin-like growth factor binding protein-7 [IGFBP7], and urinary miR-452 was significantly positively correlated with SCr, suggested that urine miR-452 may be an effective biomarker for early detection of AKI in septic patients (Liu et al., 2020c). Septic patients with AKI also had significantly lower urinary miR-376b than septic patients without AKI, and urinary miR-376b was negatively correlated with Scr and BUN in septic patients, supporting its diagnostic value for septic AKI (Liu et al., 2020d).

The expression of lncRNA TCONS_00016233 is increased in the plasma of sepsis patients. The plasma TCONS_00016233 has been shown to be positively correlated with serum creatinine, TIMP2, IGFBP7, IL-1β, TNF-α, C-reactive protein (CRP), and urinary TCONS_00016233, thus indicating that TCONS_00016233 might serve as an early diagnosis marker for the septic AKI (Zhang et al., 2020a). As mentioned above, lncRNA TapSAKI have been confirmed to be differentially expressed in sepsis rats. Previous study has found that in patients with AKI, the circulating concentration of TapSAKI is an independent predictor of patients’ survival. Therefore, TapSAKI may become prognostic indicator of SA-AKI (Lorenzen et al., 2015).

These studies demonstrated that many ncRNAs are closely related to the occurrence and prognosis of SA-AKI and have potential predictive value (Table 1). However, whether ncRNAs could be used as clinical test index still requires extensive evaluation.

**TABLE 1:** ncRNAs as potential biomarkers of SA-AKI.

ncRNA	Level	Sample	Association with Biomarker
miR-210	upregulation	serum	BUN, Cr, Cys-C
miR-49	upregulation	serum	BUN, Cr, Cys-C
miR-205	downregulation	serum	BUN, Cr, Cys-C
miR-106a	upregulation	serum	TNF-α, IL-1β, IL-6
miR-29a	upregulation	serum	Scr, Cys-C, KIM-1
miR-10a-5p	upregulation	serum	Scr, Cys-C, KIM-1
miR-26b	upregulation	urine	—
miR-452	upregulation	urine	Scr, [TIMP2]* [IGFBP7]
miR-376b	downregulation	urine	Scr,BUN
lncRNA TCONS_00016233	upregulation	serum	SCr, TIMP2, IGFBP7, IL-1β, TNF-α, CRP
		urine
lncRNA NEAT1	upregulation	serum	TNF-α, IL-1β, IL-6, IL-8, IL-10
lncRNA TapSAKI	upregulation	serum	—

### Therapeutic Potential

Correlation evidence revealed that kidney damage induced by sepsis could be improved by changing the expression of ncRNAs. Changing the expression of miR-93-5p, miR-126, miR-125b, miR-34a, and miR-155 can alleviate multiple organ damage, vascular leakage, inflammation, and cell apoptosis caused by sepsis, which indicates that changing the expression of miRNAs may become one of the crucial means to treat SA-AKI (Fan et al., 2014; He et al., 2020b). It has also been found that propofol, dexmedetomidine Ginkgolide A and dihydromyricetin reduce SA-AKI by regulating the miRNAs expression (Zheng et al., 2018; Ni et al., 2020; Li et al., 2021c; Tian et al., 2021). Moreover, another study found that curcumin alleviates renal function by suppressing lncRNA PVT1 in mice (Huang et al., 2020c). Resveratrol attenuated inflammation of SA-AKI by regulating lncRNA MALAT1 (Wang et al., 2021b). These studies have shown that changing the expression of ncRNAs can alleviate SA-AKI and can be used as a potential treatment for SA-AKI.

Gene therapy has become well accepted in clinical practice, while the related studies on SA-AKI are still limited. At present, gene therapy for SA-AKI is mainly used in animal model experiments by injecting the ncRNA’s siRNA or cDNA. However, this treatment method may become a major trend in the future and provide a new treatment strategy for SA-AKI.

## Conclusion and Prospects

The current research on ncRNA-mediated SA-AKI has focused on miRNA. The role of ncRNA in the underlying mechanism of SA-AKI is being gradually discovered, and multitarget inhibition or overexpression has been shown to have significant beneficial results in animal models or cell models. Experiments have shown that SA-AKI can be improved by changing the expression of ncRNA with drugs. However, when translated into a clinical setting, these therapeutic methods face obstacles such as inadequate safety or efficiency, which should be addressed by future studies. Meanwhile, in SA-AKI patients and animal models, some ncRNAs expressions are associated with disease severity and prognosis, and serve as independent predictors of patient survival. Therefore, the ncRNAs have excellent prospects as disease biomarkers. However, ncRNA as a clinical trial indicator still needs to be widely evaluated.
